# Considerations for the design and conduct of human gut microbiota intervention studies relating to foods

**DOI:** 10.1007/s00394-020-02232-1

**Published:** 2020-04-03

**Authors:** J. R. Swann, M. Rajilic-Stojanovic, A. Salonen, O. Sakwinska, C. Gill, A. Meynier, P. Fança-Berthon, B. Schelkle, N. Segata, C. Shortt, K. Tuohy, O. Hasselwander

**Affiliations:** 1 Division of Integrative Systems Medicine and Digestive Diseases, Imperial College London, London, UK; 2 School of Human Development and Health, Faculty of Medicine, University of Southampton, Southampton, UK; 3 Department for Biochemical Engineering and Biotechnology, Faculty of Technology and Metallurgy, University of Belgrade, Belgrade, Serbia; 4 Human Microbiome Research Program, Faculty of Medicine, University of Helsinki, Helsinki, Finland; 5 Société Des Produits Nestlé S.A, Nestlé Research, Lausanne, Switzerland; 6 Nutrition Innovation Centre for Food and Health, Centre for Molecular Biosciences, Ulster University, Londonderry, Northern Ireland, UK; 7 Mondelēz France R&D, Saclay, France; 8 Naturex SA, Avignon, France; 9 ILSI Europe, Brussels, Belgium; 10 Department CIBIO, University of Trento, Trento, Italy; 11 Johnson & Johnson Consumer Services EAME Ltd., Foundation Park, Maidenhead, UK; 12 Department of Food Quality and Nutrition, Research and Innovation Centre, Fondazione Edmund Mach, Trento, Italy; 13 DuPont Nutrition and Biosciences, c/o Danisco (UK) Limited, Reigate, UK

**Keywords:** Microbiota, Microbiome, Gut bacteria, Intestinal microbes, Human studies, Foods, Prebiotics, Probiotics

## Abstract

With the growing appreciation for the influence of the intestinal microbiota on human health, there is increasing motivation to design and refine interventions to promote favorable shifts in the microbiota and their interactions with the host. Technological advances have improved our understanding and ability to measure this indigenous population and the impact of such interventions. However, the rapid growth and evolution of the field, as well as the diversity of methods used, parameters measured and populations studied, make it difficult to interpret the significance of the findings and translate their outcomes to the wider population. This can prevent comparisons across studies and hinder the drawing of appropriate conclusions. This review outlines considerations to facilitate the design, implementation and interpretation of human gut microbiota intervention studies relating to foods based upon our current understanding of the intestinal microbiota, its functionality and interactions with the human host. This includes parameters associated with study design, eligibility criteria, statistical considerations, characterization of products and the measurement of compliance. Methodologies and markers to assess compositional and functional changes in the microbiota, following interventions are discussed in addition to approaches to assess changes in microbiota–host interactions and host responses. Last, EU legislative aspects in relation to foods and health claims are presented. While it is appreciated that the field of gastrointestinal microbiology is rapidly evolving, such guidance will assist in the design and interpretation of human gut microbiota interventional studies relating to foods.

## Introduction

It is well established that the gut microbiota is a significant variable in defining host health. Aberrant microbial compositions and/or functions have also been implicated in various disease states. As such, there is an increasing drive to design and develop novel interventions to modulate this intestinal community to promote beneficial microbial–host interactions and/or attenuate adverse outcomes. Attempting to shape the microbiota to enhance host health is not a novel concept. However, advancements in science and technology have enabled the multiple components of this expansive microbial–host relationship to be studied at high resolution [1]. This has significantly advanced our understanding of its influence on host health and our ability to evaluate the impact of interventions on this pan-organismal system. Different strengths and limitations accompany the various methodological approaches and caution must be exercised when drawing conclusions from study findings and comparing across studies and interventions [2]. Another issue is the predominance of case–control studies, which by their nature generate correlations but do not provide evidence of biological relatedness. An increased focus on intervention studies using diet to mediate specific changes within the microbiota or metabolite production linked to host physiology is needed to advance the field. Given the large inter-individual variation that exists across the gut microbiota and the numerous selection pressures on these microbes, care must also be taken when selecting target populations for intervention studies and when extrapolating the relevance of research findings to the wider population [3]. This review seeks to provide guidance to facilitate the design, implementation and interpretation of human gut microbiota intervention studies relating to foods. The focus is exclusively on human studies.

## Study design

General guidelines focusing on the design, conduct and reporting of human intervention studies to evaluate health benefits of foods [4] or for the scientific substantiation of health claims in relation to foods and food constituents are provided elsewhere [5]. Here, we focus on specific elements of study design for human gut microbiota studies relating to foods.

Research questions should be clearly stated at the outset as these will directly influence all critical aspects of the study design. Research questions of particular relevance include the following:

- What effect does the gut microbiota have on the metabolism and bioavailability of nutrients and non-nutrients present in foods?
- What effects do diets, foods or food constituents have on gut microbial composition and/or activity?
- What effects do changes in the gut microbiota composition and/or activity following a dietary intervention have on human health or on a biomarker of human health?

Although the above research questions can overlap, they help to define the study hypothesis and primary outcome.

One specific challenge for establishing if changes in gut microbial composition and/or activity have an effect on human health or biomarkers of human health is that effects on human health may be parallel but independent of changes in the gut microbiota. For example, it has been shown that the improvement in vascular function associated with cocoa flavanol consumption is predominantly attributable to the presence of the monomer epicatechin and not with the more abundant procyanidins and gut microbiome–derived cocoa flavanol catabolites [6]. Hence, such intervention studies may have limitations in determining causality because effects of dietary intervention on the gut microbiota and health outcomes may be unrelated. Nevertheless, these studies can yield relevant mechanistic insights, and together with evidence from other studies, including animal studies, can help to unravel cause–effect relationships between dietary intake and health.

Initial exploratory studies, including single arm studies, may be useful to gather information on specific aspects relating to the intervention. While the findings of such studies are unlikely to be publishable, especially single arm studies that lack an appropriate control, they do provide information that allows for better design of subsequent more rigorous randomized controlled trials (RCTs). For example, exploratory studies can help with dose-finding and provide information on the variability and size of outcome measures, information necessary for sample size calculations [4]. In the context of microbiota interventions, an exploratory study could also be used to establish if the abundance of a particular taxonomic group of interest persists after the end of an intervention and if so, for how long. Such persistence could result in significant carry-over effects in crossover studies beyond the duration of the washout period integrated in the study design, and this should be investigated by carry-over analysis of genus abundances profiles [5, 7]. Washout periods should be adjusted accordingly.

RCTs share key features such as randomization, blinding, homogenization of procedures across the study groups except for the test and control products, intention-to-treat analysis and analysis of the magnitude of difference in pre-defined endpoints [8]. Double-blind, placebo-controlled RCTs are considered as the gold standard and are preferred as the most rigorous approach [5, 8]. In general, for RCTs either parallel or crossover designs can be considered suitable for human gut microbiota studies [7, 9, 10]. Cross-over studies have an advantage of using each subject as their own control ensuring that there are no inherent differences in microbial composition and other parameters between treatment groups at baseline [9, 10]. As a result, sample sizes can be smaller. This design also enables factors contributing to responder/non-responder status to be investigated including whether it is specific to a diet or a general feature of an individual’s microbiota. This was well illustrated in a study by Salonen et al*.* [11] exploring microbial responses to three fully controlled test diets. Diet-induced changes were observed in the microbiota of some individuals (responders), while the microbiota remained stable in others (non-responders) following a dietary switch [11]. Although parallel design studies require larger sample sizes to overcome this issue of inter-individual variation in the gut microbiota, they benefit from shorter study durations requiring less commitment from study participants [5]. Furthermore, data analysis can be less complex for parallel-arm studies compared to those with a cross-over design.

## Eligibility criteria, baseline microbiota and important confounding variables in gut microbiota studies

Eligibility (inclusion/exclusion) criteria are physiological or clinical characteristics, or demographic variables used to define the study population [12]. Specific attention should be given to the following criteria known to impact on the gut microbiota.

### Age, diet and lifestyle

Certain discrete age ranges should be considered for human gut intervention studies (e.g. infants, adults or elderly) as known differences in the composition of the gut microbiota are present at different ages. The infant gut microbiota is characterized by a degree of instability with diet being one of the main drivers shaping the microbiota during infancy (e.g. breast-fed vs formula-fed) [13]. Delivery mode (vaginal birth, caesarean delivery) and gestational age are also important factors determining gut microbial composition in early-life [14, 15]. Another major event that causes dramatic diversification of the infant microbiota is weaning, i.e. the introduction of a varied solid food diet. There is still no general agreement as to when the microbiota becomes fully adult-like. Some authors report that this occurs at three years of age [16] while others have shown variation in the microbiota persists between adolescents and adults [17]. It has also been shown that the microbiota of older individuals (those above the age of 65 years) is distinct from younger adults in both composition and function [18]. The impact of interventions on microbial–host interactions and their downstream effects on health may, therefore, differ between different age groups [19, 20].

The most important restriction with regard to diet in microbiota studies is the use of supplements or products that contain prebiotics and/or probiotics. Another parameter to consider is the habitual composition of the diet, especially the dietary fiber intake. Several studies have shown that the amount of dietary fiber consumed at baseline influences the responsiveness to interventions. In a study by Healey et al*.* responsiveness to insulin supplementation (16 g/days) in healthy subjects differed, with regard to changes in the composition of the fecal microbiota, based upon their habitual dietary fiber intake (two groups, 18 g/days versus 39 g/days) [21]. As such, habitual fiber intake should be considered in the design of the human studies. Correlations were also observed between the baseline level of bifidobacteria and their growth in response to inulin, with greater growth seen in those individuals harboring lower initial levels. This is consistent with the results from Tap et al*.* where participants with a higher baseline microbial richness had gut microbiota that were more resilient to change and, therefore, less responsive to changes in dietary fiber intake [22]. In addition, many plant foods, such as artichokes and soy, contain components with prebiotic properties. The potential for the habitual diet to modulate the microbiota should be considered where possible.

Participants should be instructed not to change their principal diet and calorie intake during a gut microbiota intervention study as this may cause microbial changes unrelated to the intervention. For example, calorie restriction and other weight loss diets have been reported to change the microbiota [23, 24]; hence, exclusion of subjects on weight loss programs should be considered. The same applies to participants on other types of special diets such as vegan or diets with intention to use certain limited food groups only (e.g. paleo diet, gluten-free), although this will be study-specific depending upon the research question. Hence, habitual diet should be taken into account at least when analyzing the data as a potential confounder or mediator of the intervention effect [4, 5].

Measurement of food intake combined with food composition is key to assessing the impact of food on health. However, assessing dietary intake is a major research challenge [25]. It is important to note that all self-reporting methods have error, such as random day to day variability and systematic error or bias. Understanding the nature of the error can ensure better assessments and interpretation of results. As such, there is a longstanding desire to identify biomarkers that provide objective measures of dietary intake with minimal error. However, to date few exist other than bodyweight, doubly labelled water, 24 h nitrogen, urea, potassium and sodium [26] and a selection of metabolic markers indicative of specific nutritional intake (e.g. urinary proline betaine for citrus fruit intake) [27]. Metabolomics, metagenomics and natural enrichment of stable isotopes are being increasingly used to identify novel biomarkers or methods to assess intake [28, 29]. Ultimately, the main research question of the study will dictate the dietary methods chosen to characterize/ the main dietary component(s) of interest and a Best Practice Guideline exists that outlines the questions that should be addressed to determine the most appropriate dietary tool to use [30].

Recent studies, both in laboratory animals and in humans. have suggested gender differences may exist in gut microbial composition [31, 32]. Moreover, microbiota response to dietary components has also been suggested to differ between men and women [33]. Such variation could contribute to sex differences in vascular, metabolic and immune parameters apparent in some dietary interventions [34, 35]. However, few human dietary interventions have been conducted with sufficient statistical power to delineate sex-specific microbiota responses. Potential gender effects could have significant implications for future dietary interventions, suggesting much larger sample sizes may be required to account for different effect sizes in men and women. Similarly, possible sex effects could have implications for previous dietary interventions which failed to show positive changes in microbiota or related physiological responses, since failure to take sex bias into account when calculating sample sizes may have led to the statistical underpowering of studies and possible false negatives [36].

Exercise level is another lifestyle factor known to impact on gut microbial composition [37] and participants planning on changing their exercise levels or intensity should be excluded. Long-distance travel and its associated jet lag have also been identified to lead to aberrant microbiota diurnal fluctuations [38, 39].

### Baseline microbial composition

Health status and drug/medication use are important parameters to record to aid interpretation of results. Despite an accurate a priori definition of the study population based on specific eligibility criteria, significant variation of the microbiota across study participants cannot be avoided [40]. In the future, inclusion and exclusion criteria may incorporate the baseline microbiota profile to ensure that study groups are comparable and eventually for optimizing intervention efficacy. This may include, for example, measuring microbiota richness or diversity, clustering within specific enterotypes or targeting a specific bacterial group if it is expected to be involved in the mechanism of action. Multiple measurements within the same individual may be necessary to provide an accurate understanding of the individual’s microbiota, e.g., by including microbiota analysis before and after a 2-week run-in period [7]; however, this may not always be practically feasible.

### Environmental considerations

While it may not be feasible to incorporate truly environmental factors into the inclusion/exclusion criteria it may be beneficial to record such information to assist the interpretation of results.

The microbiota of healthy adults is thought to be relatively stable and resistant to colonization by both pathogens and commensal microbes, as confirmed by metagenomic analyses showing that ca. 70% of the strain-level signature of the adult microbiota is constant in time [41]. In contrast, in early life, during age-driven microbiota succession there is a high turnover in species [14]. Due to the adaptation of the intestinal microbes to gut environment, social-environmental conditions, such as crowding, family composition and family size are likely to influence the gut microbiota, especially in early life. Family members share bacterial strains, implying transmission among family members has a strong influence on shaping gut microbiota [42]. For example, Sjogren et al*.* showed that the number of family members is positively correlated with the number of bifidobacteria species [43]. Household pets can be a particularly rich source of microorganisms, especially for children [44], where it has been reported that infants raised in households with dogs harbored more *Ruminococcus* and *Oscillospira* [45]. Hygiene practices within households and the use of anti-microbial products is also likely to influence microbial exposure and transmission. However, capturing such information in an objective manner that can inform participant inclusion/exclusion is likely to be difficult.

### Sample sizes

Calculating the number of participants required for microbiota intervention studies is challenging for several reasons. In many cases it is not possible to select beforehand, which specific bacterial taxa are expected to be modulated by the particular dietary intervention in a given population. Another issue is that it is still not possible to define a normal (eubiosis) or abnormal microbiota (dysbiosis). Hence, it is difficult to interpret an increase or decrease of specific microbiota groups as an indication of any specific health effect. Unsurprisingly, the extent of changes in microbiome composition and structure that could be considered biologically relevant is difficult to determine. The current EFSA guidance document [46] does not specify any biologically relevant effect sizes (after public consultation of the earlier 2011 version [47], the suggestion of at least 1 log decrease of detrimental organisms was dropped from the final document) [48]. Some inferences regarding relevant effect sizes could possibly be made from studies comparing microbiota in subjects with different health status. For example, it could be argued that an increase of approximately 0.5 units of Shannon diversity index may be biologically beneficial, as this is the difference observed between obese and lean individuals [49]. Consequently, it is more likely that study group size is defined based on other measured parameters, such as biochemical and clinical traits.

The outcome measure used to calculate the necessary sample size will depend on the hypothesis being tested. If measurement of bifidogenesis upon ingestion of a test fiber is sufficient to establish selective fermentation in vivo and possible prebiotic status, then sample size calculations could be performed using effect sizes and standard deviations for fecal bifidobacteria measured quantitatively using quantitative polymerase chain reaction (qPCR) or fluorescence in situ hybridization (FISH) for example. If, on the other hand, a trial is designed to test whether ingestion of a particular probiotic strain impacts on blood cholesterol levels, then cholesterol lowering should be selected as primary objective and data from existing similar studies should be used to design a trial with sufficient statistical power to demonstrate a cause and effect relationship between ingestion of the test probiotic and this recognized health effect.

## Characterization of the product/foods

The product evaluated in microbiota intervention studies must be well characterized. In addition to the macronutrient composition (fat, protein, carbohydrates), focus should be on components that possibly act as microbial modulators including fibers, certain micronutrients as well as non-nutritive compounds such as certain polyphenols or probiotic cultures. The following describes the specific characterization requirements of known microbial modulators such as probiotics, prebiotic fibers and polyphenols, which can be provided in purified form.

Probiotics are defined as live microorganisms that, when administered in adequate amounts, confer a health benefit on the host [50, 51]. Correct identification and characterization of such live microorganisms (e.g. bacteria and yeast) is considered of critical importance by Regulators in particular if endpoints from a probiotic intervention study are provided as evidence to support health claim applications. This is important as observed physiological effects in the host are species- or even strain specific [52]. Strains should be named in accordance with the International Code of Nomenclature (for bacteria according to the International Committee on Systematics of Prokaryotes (https://icsp.org/), and for fungi according to the International Code of Nomenclature by the International Commission on the Taxonomy of Fungi (ICTF) (www.fungaltaxonomy.org)) or MycoBank (https://www.mycobank.org). It is recommended to deposit strains in an internationally recognized culture collection (https://www.wfcc.info/collections/).

Before studying health benefits associated with probiotics in vivo, probiotic candidate strains should be characterized in vitro. Phenotypic and physiological properties such as carbohydrate fermentation, enzymatic activity and in particular, ability to survive and grow under conditions similar to those in the GI tract should be determined. In addition, safety of the probiotic must be demonstrated either by a history of safe use and/or specific in vitro and in vivo safety assessment prior to use in a human intervention study. Viability and stability considerations are also critical for probiotic interventions. Viable cell count and stability of count for the duration of the human intervention study under defined storage conditions and in the corresponding matrix/delivery format should be monitored and documented. While viable cell number has been shown to impact probiotic efficacy [53], it should be noted that there are examples of probiotics that convey beneficial health effects even in pasteurized form [54].

A prebiotic is defined as a substrate that is selectively used by host microorganisms conferring a health benefit [55]. This updated definition has expanded the description of a prebiotic to other food components such as polyphenols and fatty acids in addition to oligosaccharides (e.g. fructooligosaccharides, galactooligosaccharides and human milk oligosaccharides) and dietary fibers. Dietary fibers are carbohydrate polymers with three or more monosaccharide units, which are neither digested nor absorbed in the small intestine. They belong to the following categories—edible carbohydrate polymers naturally occurring in the food as consumed;edible carbohydrate polymers that have been obtained from food raw material by physical, enzymatic or chemical means and which have a beneficial physiological effect demonstrated by generally accepted scientific evidence [56]. If isolated fibers or prebiotic compositions are tested in a microbiota intervention study, the following parameters could be considered for characterization: Source of fiber; carbohydrate/sugar composition; purity of fiber including residual mono- and disaccharides that may be present in the fiber preparation; degree of polymerization, average molecular weight and molecular weight distribution range and glyosidic linkages. Solubility and viscosity as well as data supporting resistance to digestion should be available.

To identify a molecule as a fiber, resistance to digestion must be demonstrated. A common in vitro assay used to determine total dietary fiber content is the AOAC method 2009.01 [57], which uses pancreatic α-amylase and amyloglucosidase to mimic carbohydrate digestion. However, the absence of brush border enzymes in this assay is a limitation. This is particularly true for glucose-based *α*-glycosidic-linked oligomeric dietary fiber candidates whose in vivo digestibility is poorly predicted. Digestibility assessments (glucose response) in mice or humans may be warranted in such cases [58].

In their glycosylated and polymeric forms, polyphenols are usually poorly absorbed in the upper gut and pass into the large intestine where they can act as microbial modulators [59]. If isolated polyphenol preparations are used in a microbiome intervention study, the source of polyphenols should be described and their chemical structure should be characterized including oligo-/polymer content and degree of polymerization. The amount of polyphenol in an intervention can be underestimated as some ingredients and foods, depending on the matrix (whole fruit, fruit powder, plant or fruit extracts) and the process used to prepare food or ingredients, can also contain polyphenols. Non-extractable polyphenols (NEPP) include all those phenolic compounds that are not extracted with the solvents of choice and, therefore, are not assessed in most polyphenol analyses [60]. NEPP consist of polyphenols that belong to different classes, such as macromolecular polyphenols or single polyphenols associated with cell wall macromolecules [61]. A recent re-evaluation of the polyphenol content of different food products has demonstrated that NEPP could represent a significant proportion of the total polyphenol content of food and may be considered as the major fraction of plant food polyphenols in some cases [62, 63]. These NEPP are not absorbed in the small intestine, reach the colon together with fiber and other undigestible constituents and can be extensively metabolized by the gut microbiota into smaller phenolic compounds that are better absorbed and could persist in blood circulation until 48 h after consumption.

In addition to the general requirements for a suitable control (e.g., matched to the test product in terms of sensory characteristics, appearance, energy content, nutrient composition and route of administration), the placebo/control product should be selected on the basis of having a minimal effect on microbiota composition and/or activity. Maltodextrin is frequently used as control product in prebiotic interventions, but there is some evidence that maltodextrin can alter microbiota composition and increase colonic volume when used at high doses [64]. Microcrystalline cellulose could be used as a control in interventions where isolated dietary fibers are studied, as it contains minimal energy and is less fermented by gut microbes compared to alternative control fibers [65].

## Compliance

The validity and relevance of any intervention study depends on a high level of participant compliance, i.e., the degree with which a study subject adheres to the experimental protocol. Hence, factors affecting compliance should be considered when designing the study to maximize protocol-related compliance. Assessment of compliance with dietary interventions is necessary to understand the observed magnitude of effect of the intervention*.* Hence, methods for measuring and improving compliance should be implemented. Factors affecting compliance in intervention studies have been covered extensively previously [4].

Specific additional factors that may impact compliance in human gut microbiota intervention studies include the sampling burden, particularly, fecal sampling and storage of fecal samples by participants at their home. In most microbiota studies involving collection of fecal samples, participants are required to store samples in their home freezers immediately and then bring them to their next study appointment or collection point. At present, there is little information available how sampling aversion may affect study compliance. A survey conducted as part of the Flemish Gut Flora Project indicated that only 5% of subjects who completed the full protocol considered quitting because of sampling aversion and only 2.8% of respondents indicated that the requirement to store fecal samples in their home freezer was the reason to quit participation. The most important elements of the fecal sampling procedure captured from the survey were clear sampling manuals, sampling hygiene and ease of sample handling [66]. Although the overall drop-out rate appeared to be low due to sample aversion and home storage requirements, it seems prudent to provide suitable kits with clear instructions and easy handling.

Specific biomarkers for intake, e.g., analysis of blood, urine or fecal metabolites can be used as an independent and objective measure of compliance and these should be used if possible.

The presence of specific probiotic strain(s) used in the intervention can be determined in fecal samples collected during and at the end of an intervention and compared to baseline samples collected prior to intervention. This can be achieved using targeted qPCR analysis.

Presence of alkylresorcinol metabolites in urine can correlate with the intake of whole grains and cereal brans (particularly rye and wheat) and can be used as a short/medium-term biomarker in such interventions [67]. However, phenolic compounds can be lost in purified fibers. Urinary or fecal ferulic acid or serum dihydroferulic acid have also been described as possible markers for rye bran or whole-grain wheat intake [68, 69].

Increase in fecal short-chain fatty acids or decrease in fecal pH at the end of the study compared to baseline sample prior to intervention can be an indicator of fermentable fiber intake. However, these are related to microbial activity and variation in these measures could reflect variation in microbial response to these fibers.

Depending on the type of polyphenol used in the intervention, blood, urinary or fecal biomarkers could be used to measure compliance. For polyphenols with low bioavailability (e.g., conjugated or polymeric polyphenol forms) that cannot be measured directly in blood samples, the principal microbial metabolites described by [59] could be used to monitor compliance. One should also consider that the gut microbiota shows high inter-individual variability for its capacity to produce different types of metabolites from the same family of polyphenols. Indeed, study participants could be stratified by their polyphenol-metabolizing phenotypes producing different types of active metabolites responsible for the health benefits. These metabotypes could reflect the gut microbiota composition and metabolic status and become markers for gut-microbiota related polyphenols health benefits [70].

Defining acceptable levels of compliance should be done a priori and a major deviation should result in the exclusion of non-compliant subjects.

## Outcome assessment

To demonstrate an intervention can confer health benefits to the host via interactions with the microbiota, both microbiota- and host-specific assessments should be made. A number of parameters can be measured, and the metric studied is dependent on the type of intervention used and its intended effect. Such assessments can evaluate the microbial landscape (typically bacterial cell populations, composition/abundance or its genetic content/metabolic potential) and/or the biochemical output of the microbiome and its metabolic exchange with the host. Health benefits can occur not only through immunological and biochemical mechanisms but also through more complex interactions along gut-brain axis. Understanding microbiota composition is particularly important from an immunological perspective while characterizing microbial function is important to understand the metabolic capacity of the microbiota and the downstream impact of its metabolites on host physiology and biochemistry. Host-specific measures should also be assessed providing a functional endpoint related to health. Such measurements can be diverse and are directed by the anticipated outcome of the intervention. Below is a summary of endpoints or markers that have been used to demonstrate a microbial effect on host health (summarized in Table 1).

**Table 1:** Examples of bacterial, host, outcome markers that have been used in studies

Endpoint/marker	Type	Sample type	Associated health benefit
Change in abundance of lactobacilli	Bacteria	Feces	Compliance for probiotic supplementation, probiotic activity if linked to health outcome
Change in abundance of bifidobacteria (bifidogenic effect)	Bacteria	Feces	Compliance for probiotic supplementation, probiotic activity if linked to health outcome
Effect on gut commensals e.g.,* Akkermansia muciniphila*, *Prevotella copri, Faecalibacterium prausnitzii*, *Prevotella*-to-*Bacteroides* ratio, Christensenella, *Faecalibacterium* and *Coprococcus*	Bacteria	Feces	Various health outcomes such as metabolic health, gut and immune health, brain health
Alpha-diversity: Microbiota richness and evenness in a specific sample	Bacteria	Feces	
Beta-diversity: Heterogeneity of the microbiota among the analyzed samples	Bacteria	Feces	
Gene richness	Bacteria	Feces	Metabolic health
Lipopolysaccharide (LPS)	Bacterial cell wall component	Blood	Translocation, inflammation, impaired barrier function
LPS-binding protein	Host protein	Blood	LPS exposure, translocation, impaired barrier function
Inflammatory cytokines (including. IL-1β TNF-α, IL-6, IL-12)	Cytokine	Blood, mucosa	Inflammatory cytokines elevated in IBD and gut inflammation
Acetate	Metabolite	Urine, blood, feces	Reduced intestinal pH, pathogen exclusion, mineral absorption, appetite regulation, energy source, alters intestinal motility
Butyrate	Metabolite	Urine, blood, feces	Reduced intestinal pH, pathogen exclusion, mineral absorption, enterocyte energy source, stimulates apoptosis, glutathione regulation, modifies tight junction permeability, alters intestinal motility, anti-inflammatory properties in colonocytes, stimulates gut mucin production
Propionate	Metabolite	Urine, blood, feces	Reduced intestinal pH, pathogen exclusion, mineral absorption, appetite regulation, improves insulin sensitivity and glucose tolerance and modifies lipid metabolism, hepatic gluconeogenesis, alters intestinal motility
Succinate	Metabolite	Urine, blood	Inhibits hepatic glucose output to improve glucose and energy metabolism
Imidazole propionate	Metabolite	Blood	Impairs glucose tolerance and insulin signaling by activation of mTORC1
Hydrogen sulfide (H_2_S)	Metabolite	Feces	DNA damage, intestinal inflammation
Polyamines (agmatine, tyramine, histamine, cadaverine, putrescine, spermidine, spermine)	Metabolite	Urine, blood, feces	Immunoregulatory effects, oxidative stress, inflammation, genotoxicity
Chenodeoxycholic acid	Metabolite	Urine, blood, feces	Thermogenesis (energy expenditure) in brown adipose tissue
Secondary bile acids	Metabolite	Urine, blood, feces	Cholesterol gallstone formation, colorectal cancer, modifies *C. difficile* infection susceptibility, lipid digestion/absorption, metabolic signaling/ regulation
Indole and indoxyl-sulfate	Metabolite	Urine, plasma	Gut microbial proteolysis markers, aryl-hydrocarbon receptor (AHR) ligands,
4-Cresyl sulfate (*p*-cresyl sulfate)	Metabolite	Urine	Gut microbial proteolysis, genotoxicity, impact on microbiota diversity, modulates host phase II drug metabolism
Hippurate	Metabolite	Urine	Inversely correlated with blood pressure and BMI, marker of microbial polyphenol metabolism
Trimethylamine-*N*-oxide (TMAO)	Metabolite	Urine and blood	Associated with CVD, possible marker of renal function

### Assessing microbiota composition and diversity

Molecular approaches are typically used to study the microbiota primarily based on the sequence of the phylogenetic marker—small subunit ribosomal RNA (SSU rRNA) sequence (16S in prokaryotes and 18S ribosomal RNA in eukaryotes) [71]. Currently, the most commonly used method for the assessment of gut microbiota composition and diversity is sequencing of *SSU rRNA* gene coding fragments. This approach is typically referred to as 16S rRNA gene sequencing, while next generation sequencing (NGS) is a general term that in microbiota studies can refer either to targeted amplicon sequencing or shotgun sequencing [72]. NGS enables the comprehensive analysis of the microbiota and generates an output that can be interpreted at various levels. Sequencing data are typically summarized into bins of highly similar sequences, operational taxonomic units (OTUs). Based on the number of reads of OTUs various analyses can be performed including calculation of diversity indices (α and β), semi-quantitative analysis, multivariate statistical analysis and correlation with host or environmental factors. NGS is by far the most applied approach in microbiota studies. However, it has some disadvantages, particularly for intervention studies. One major drawback is that the read length for NGS is short (typically 2 × 250–300 bp for Illumina) and only a portion of the *SSU rRNA* gene can be read, causing a loss of sensitivity (most taxa cannot be reliably defined at the species level, although high confidence identification of higher taxonomic rank is possible) [73]. Even when performed at significant sequencing depth, NGS provides information only about predominant taxa in the ecosystem, while species of interest in an intervention study might be subdominant and therefore, below the detection threshold of sequencing. Alternatively, targeted methods that use optimized regions of the *SSU rRNA* gene or other genomic regions that provide higher resolution for the taxon of interest can be used to quantify specific groups of organisms (typically genus or species members) in complex samples. These methods include fluorescent in situ hybridization (FISH) analysis and quantitative polymerase chain reaction (qPCR). FISH is the only method that allows direct visualization of histological localization of microbes in the tissue and thus gives an insight into correlation between microbial presence and any observed histopathological changes. The usefulness of this technique was demonstrated in one animal study showing that certain dietary emulsifiers could alter mucus structures allowing gut bacteria to penetrate deeper into the mucus layer and closer to the epithelial cells, which

induced low-grade inflammation [74]. A limiting technical demand of FISH analysis is that microbial cells have to be fixed prior to analysis. This means that samples have to be pre-prepared while still fresh. qPCR is a DNA based method that allows a rapid and sensitive method of detection and quantification of microbial groups at various levels of taxonomic resolution [75]. Analysis of microbiota by qPCR is particularly convenient if one aims to measure the effect of a dietary intervention on a specific microbial group of interest, e.g., for assessing the abundance of typical probiotics—*Bifidobacterium* and *Lactobacillus*—during dietary intervention. Moreover, both FISH and qPCR are quantitative methods, enumerating microorganisms directly in situ or indirectly by measuring *SSU rRNA* copy number. Such stand-alone, quantitative data have advantages, especially when being correlated with other absolute data compared to relative abundances typically used in metataxonomics. The most comprehensive pre-designed methods for microbiota assessment are microarrays (DNA arrays). Microarrays are based on simultaneous hybridization of thousands of molecular probes with nucleic acid material from a complex microbiota sample. Several phylogenetic microarrays have been developed for the analysis of the human gut microbiota [18, 76, 77]. Microarrays provide a general overview of the ecosystem response and enable the sensitive detection of intervention probiotic species. This can include stimulated and suppressed microbial groups as was demonstrated with inulin-type fructans in obese women [78]. The major limitation of microarrays is the incomplete coverage of the ecosystem (that still has not been described) while the advantages include high reproducibility, simultaneous identification and quantification. Particularly because of the high reproducibility and similar and comparable output of the analysis of a large number of samples, microarrays are a useful method for defining (complex) microbiota signatures that are relevant for a dietary treatment, as exemplified by the microbiota-based definition of responders to the low FODMAP diet [79]. In the area of microbiota analysis, technological developments are rapid, and novel or improved versions of the existing methods emerge regularly. Furthermore, no consensus regarding the use of one particular methodology for assessing gut microbiota resulting in highly variable datasets in the literature, which are often difficult to compare.

Metagenomics is a powerful integrative approach that enables analysis of microbiome functional potential and diversity based on sequencing of the total genomic material of intestinal samples [80]. This can yield information about specific functional shifts in addition to species- and even up to strain-level variation, which is particularly relevant given the high functional redundancy of the gut microbiome [81], whereby different microbes can perform the same function [82]. Due to the high analytical costs and computational needs, metagenomics has not yet been widely applied in dietary intervention studies. However, it has been applied for predicting personalized postprandial glycemic responses [83], for studying the effects of probiotic versus flaxseed mucilage on the gut microbiota and metabolic risk markers [84], and for providing mechanistic insights into diet-microbiota–health interactions in diabetic individuals [85]. Most computational profiling tools for metagenomics are reference-based [86–88], but the lack of genomes for a large fraction of intestinal inhabitants limits their performance. Improvements of these reference databases are essential for the progress of the field, and recent work using cultivation and metagenomics assembly is supporting this. For example, the annotation of over 1500 cultured gut microbes enabled the annotation of metagenomic reads to increase from 50 to > 70% and the large-scale metagenomic reconstruction of over 150,000 microbial genomes (77% of which were from previously uncharacterized species) from a large set of metagenomic samples. This increased the median annotation rate to 95% [89], altogether facilitating higher-resolution descriptions of the human gut microbiome [90]. Finally, it should be noted that interaction between humans and their microbes is complex and that expression of particular genes does not depend solely on the available genetic portfolio, which can be assessed via metagenomic analysis.

### Considerations for microbiota analysis related to sample collection and analysis

Several comprehensive reviews on the essentials of conducting a microbiota study have been published [66, 80, 91–94]. These articles provide practical recommendations that are briefly summarized here for fecal samples, the most common sample source for intestinal microbiota in dietary intervention studies. In most clinical studies home sampling with immediate freezing is the recommended and most attainable choice for sample collection. Special care must be taken to maintain the cold chain during the transport to study center, as freeze–thaw cycles increase the risk of altering the community composition. This is especially relevant for protocols that include sample fractionation, e.g., to pellet cells before DNA extraction, as this leads to loss of cell-free DNA from bacteria that lyzed during freeze–thawing. Use of mechanical cell lysis prior to DNA isolation ensures that the more recalcitrant microbes are correctly represented in the community DNA [95]. Use of mock communities, either in th form of a bacterial cell mixture or community DNA, is highly recommended for identification of potential sources of bias, especially when setting up the analytical pipeline [95]. Although the sequencing platform used and the choice of bioinformatic tools and parameters can considerably influence the microbial profiles measured, their discussion is outside the scope of this article. For literature on this topic, please see the following articles: [92, 93, 96, 97]. While the current literature is partly inconclusive to suggest best practices for all decisions on sample handling and analysis when conducting a microbiota study, it is essential to maintain the same methodology throughout the study.

Large, population-level microbiota studies indicate that at best, *ca.* 20% of the overall microbiota variation can be explained with the commonly collected metadata, including diet, anthropometry and medication [35]. Previously ignored factors such as stool consistency, transit time [98] and timing of fecal sampling [99] are now known to influence the microbial signature of samples and hence emerge as novel sources of variation in microbiota studies. It can be argued whether these variables are more biological or technical in nature, but consideration of these factors will assist researchers in de-noising the identification of microbiota treatment effects in intervention studies.

### Considerations for bioinformatics and statistical analysis

The general data analysis pipeline for microbiota studies, which should be documented prior to initiation of the study, aims at interpreting data matrices in which measures of microbial features including taxa abundance, presence of functional markers, immunological markers and metabolites’ abundance are cross referenced with the samples in the study and its associated metadata. In several cases (e.g.,* 16S rRNA* gene sequencing) the data matrix contains count rather than continuous data with many zero values, which cannot be log-transformed to gain more normal distribution. Hence, statistical models based on negative binomial or poisson distribution should be used for reliable identification of taxa that differ in abundance between study groups [100, 101]. Typically, uneven sequencing across samples further introduces biases that should be partially tackled, e.g., by using rarefaction approaches or using read count as offset in statistical models. Sequencing-derived microbiota measurements also suffer for compositionality issues (i.e., the values are fractional, not absolute) that can generate false correlation and differential features. Different approaches, such as flow cytometry [102] and qPCR [103] have been introduced to enable quantitative microbiota profiling to complement the current practices and to specifically overcome the issues related to compositionality of relative abundance data.

Analysis approaches include unsupervised exploration of the data such as dimensionality reduction techniques, or heatmap-based and network-based visualizations that can provide indications for statistical hypothesis-testing analyses. Supervised methods are instead based on univariate or multivariate statistics with tools such as analysis of variance (ANOVA) or permutational multivariate analysis of variance (permANOVA) that can test the significance of separation between groups given a meaningful measure of inter-sample beta-diversity. Several approaches for biomarker detection and assessment are available from univariate and multivariate statistics with additional tools specifically developed for microbial data. Complementary to the statistical approach, machine learning can be used to train learning models to use the microbiota as a predictive tool and to assess the strength of association between the microbiota and conditions of interest without assumptions on the underlying distribution of the measurements.

Between group comparisons should be generally prioritized to evaluate the effects of the intervention on the microbiota, but especially in small and moderately sized parallel arm studies, the large within-group microbiota variation and/or the potential between-group microbiota difference already at baseline necessities the use of within-group (paired sample) comparisons (e.g., [104]). In this case, the baseline microbiota can and should be used as a covariate.Assessing microbial endpoints

Typical microbiota-related endpoints derived by compositional analysis in dietary intervention studies include alpha- and beta-diversity, taxa richness, and the relative abundance of individual taxa, typically genus-level and higher. However, giving biological context to any of these measured parameters is disputable. A major limitation is that the microbiota is still not fully described, and due to enormous inter-individual variability, many, even uncultured species, have not yet been detected. It has been estimated that comprehensive analysis of the gut ecosystem of over 40,000 individuals would be needed to estimate Western European microbiota richness. Nevertheless, dysbiosis, referring to disturbed gut microbiota composition, was detected in a number of diseases by comparing microbiota of patients and controls. Lower alpha-diversity, enrichment or depletion of specific bacteria and often enrichment in bacterial virulence factors can be associated with microbial dysbiosis characteristic of many chronic diseases. Based on the markers of dysbiosis it is possible, to some extent, to define desirable microbial endpoints, although none of these endpoints can be seen as an absolute marker of health, as will be discussed below.

Traditionally recognized probiotic bacteria, members of genera *Lactobacillus* and *Bifidobacterium* are perceived as beneficial for host health and their increase during an intervention is generally considered beneficial from a scientific rather than a regulatory perspective. There are many dietary interventions that showed a positive impact on the host coupled with an increase in these commensal bacteria [105]. However, there are examples of successful dietary interventions that reduced the abundance of these beneficial bacteria while achieving improvement of host health [106].

Elevations in the abundance of butyrate-producing bacteria are also generally considered beneficial for health. There are several metabolic pathways for butyrate production that can be performed by different genera belonging to Ruminococcaceae and Lachnospiraceae families [107]. Among the butyrate-producers that include, among others, *Roseburia, Anaerostipes, Coprococcus* and *Faecalibacterium* genus, the latter is widely recognized as a marker of a healthy microbiota [108]. Another more recently recognized beneficial microbe is *Akkermansia muciniphila*, particularly in obesity and metabolic disorders, although its decreased abundance has also been reported in other pathologies [109]. *Akkermansia muciniphila* is a prevalent and relatively abundant intestinal microbe of humans of different ages. It typically reaches abundance between 10^8^ and 10^9^ cells per gram of feces and represents between 1 and 4% of the total microbial community [110, 111]. Several studies have reported, using different methods, decreases in the abundance of this bacterium as BMI increases [112]. It has been shown that individuals supporting a higher abundance of *Prevotella* were in general more metabolically healthy [113].

Alpha diversity is an ecological measure that reflects the microbiota richness (number of different species or other taxonomic units) and evenness (relative abundances of different taxa). Beta-diversity reflects the heterogeneity of the microbiota among the analyzed samples and is typically calculated as the Bray–Curtis dissimilarity index that can vary between 0 (identical samples) and 1 (no compositional overlap). These endpoints are typically compared between the treatment groups and/or the timepoints (baseline and post-intervention). Their delta values can also be calculated to measure the directionality and magnitude of change. Variance partitioning, e.g., with permutational ANOVA (permANOVA) of beta-diversity provides a useful measure to quantify and rank order the sources of variation in the microbiota data to the intervention versus other effects [11].

High microbiota diversity richness, as well as the gene richness from metagenomic studies is generally considered desirable, health-related microbiota endpoint. This stems from the fact that in any ecosystem community structure and stability are generally supported by high diversity which builds in metabolic redundancy and the ability to respond to environmental challenges or stressors without disruption of community structure. However, it should be noted that infants are an exception from this general rule as, especially in breastfed infants, their microbiota is simple and dominated by a single genus—*Bifidobacterium* [114]. Although high diversity and “adult-like” microbiota in infants is associated with health risks such as atopic disease [115], the diversification of the microbiota and increase in butyrate-producers from the age of four to six months is considered developmentally appropriate and has been associated with reduced risk of asthma and wheezing [116]. This demonstrates that it is essential to take into account the distinct phases of microbiota assembly and focus on age-appropriate development.

Even in adults high diversity alone does not always reflect “health”. For example, an increase in species richness [117] has been reported in colorectal cancer patients. Rather, this should be considered along with other ecological parameters, such as structural stability over time [11, 79, 118, 119]. Diet, especially the diversity and quantities of chemically distinct substrates reaching the colon is likely to play an important role in driving and maintaining species richness and metabolic diversity within the gut microbiota. However, a reduction in fecal microbiota alpha-diversity may not necessarily reflect a deleterious impact on host health or indeed reduced microbial health service provided to the host.

### Assessing functional status of the microbiota

It is important to understand the functions performed by the microbiota. Metabolic profiling (metabolomics/metabonomics) aims to simultaneously measure several to thousands of low molecular weight metabolites present in a biological sample. These metabolites reflect the biochemical events occurring within the system from which the sample were obtained. In terms of human samples, these compounds can arise from host endogenous metabolic processes but also those of the gut microbiota, as well as the diet, and interactions between all three. Using metabolic phenotyping, the biochemical output (functionality) of the gut microbiome can be determined as well as how these outputs impact on host biochemistry. This is a powerful technique to assess the functional impact of dietary interventions on the microbiota and subsequently the host.

Nuclear magnetic resonance (NMR) spectroscopy and mass spectrometry coupled to either gas chromatography (GC–MS) or liquid chromatography (LC–MS) are typically used to measure the metabolic profiles. A range of sample types can be studied from the host including urine, blood (plasma/sera), feces, and saliva. Some studies have used intestinal biopsies and aspirate samples collected from the small intestine, but the invasiveness of their collection means that these samples are rare for intervention studies. Given their relative ease of collection urine, fecal and blood samples are typically analyzed in human studies. The fecal metabolome is thought to reflect the metabolic interplay between the diet, host and microbiota. A recent study including 786 individuals, found ~ 68% of the total variance in the fecal metabolome (based on 1116 fecal metabolites) was explained by the fecal microbial composition [120]. However, it can be argued that as fecal metabolites remain unabsorbed from the GI tract they are ‘unseen’ by the host metabolic system. As such, urine and plasma/serum samples may be more informative regarding the biochemical interactions between the microbiota and host.

### Assessing metabolite endpoints

Microbes in the gut can transform intestinal substrates into a spectrum of metabolites that can exert local effects in the gut or be absorbed from the GI environment, pass through the liver and enter the systemic circulation where they can have systemic effects. It is now well established that metabolites from the gut microbiota, as well as their structural components (e.g., bacterial lipopolysaccharide), can influence human health. It is also important to consider the kinetics of metabolite production, absorption and clearance from different tissues during nutritional metabolomics studies. Most metabolites produced by the gut microbiota from ingested foods appear in blood after only 5–7 h of ingestion, and most will be cleared from the blood rapidly, with only low levels persisting after 12–18 h. This has important implications when trying to link microbial metabolites with physiological parameters in chronic dietary interventions. In the majority of cases health parameters are measured in overnight fasted plasma/serum samples, while most microbial metabolites might be cleared from the blood in these same samples. Similarly, analytes in 24-h urine samples more accurately reflect dietary intake over the previous days than spot urine samples. Microbial metabolite kinetics is further complicated by the enterohepatic circulation. Such considerations are important when designing experimental schemes to measure microbiota metabolite production from particular foods [121] (Table 1).

SCFA are the major end-products of the bacterial fermentation of non-digestible carbohydrates and can be measured in fecal, urine and plasma samples. The main SCFAs are acetate, propionate and butyrate and these metabolites underlie many of the putative health benefits associated with the gut microbiota. SCFA have been found to modify many host processes, including various metabolic pathways, immunological functions and the expression of several genes with potential to impact on health, such as, lowering the pH of the gut [122] and the facilitation of mineral absorption [123].

Depending on their chemical structure, amino acids can be metabolized by the intestinal microbes into beneficial metabolites or converted to products potentially harmful for the host [124]. Sulfur amino acids and sulfated compounds can be metabolized to hydrogen sulfide (H_2_S), a toxic compound associated with DNA damage and intestinal inflammation [125, 126]. Bacterial decarboxylation of amino acids can produce biogenic amines and polyamines. This includes the production of agmatine, tyramine, histamine, cadaverine and putrescine (which can be further catabolized to spermidine and spermine). Polyamines are important for maintaining the structural integrity of nucleic acids and membranes as well as in gene regulation and translation while histamine can induce immunoregulatory effects [127]. However, high amounts of these metabolites have been implicated in oxidative stress, inflammation and genotoxicity [128]. Keto acids (α-keto-β-methylbutyrate, α-keto-γ-methylvalerate, α-ketocaproate, α-keto-β-methylvalerate) are produced from the bacterial degradation of branched chain amino acids and branched chain fatty acids arise exclusively from the bacterial fermentation of these products. Several of these metabolites can alter the mucosal immune system and modify signaling pathways in epithelial cells [129, 130]. Some of the microbial metabolites of tryptophan, including indole, indole-acetate and 3-methyl indole (skatole), are known ligands for the aryl-hydrocarbon receptor, as well as tryptophan itself.

Bile acids are another major class of metabolites strongly influenced by the gut microbiota. These compounds hold a key role in the digestion and absorption of lipids, nutrients and lipid-soluble vitamins. Bile acids are continually circulated between the host liver and intestinal microbes, which contribute significantly to the diversity of the bile acid pool. Microbially produced secondary bile acids are potentially more cytotoxic for the host than the primary bile acids and have been linked with cholesterol gallstone formation and colon cancer [131, 132]. However, the production of deoxycholic acid has been associated with protection against *C. difficile* infection, where patients with *C. difficile* excrete lower amounts of secondary bile acids in their feces [133]. Restoration of 7α-dehydroxylation activity, via fecal microbial transplantation, has been shown to enhance resistance to *C. difficile* [134, 135].

### Assessing immunology-related microbiota–host interactions

The gut microbiota contributes significantly to immune effector cell activity and maturation [136, 137] and its dysbiosis has been associated with autoimmune diseases including inflammatory bowel disease, food allergies and asthma [138]. The gut microbiota affects both innate and adaptive immunity through multiple mechanisms including direct contact with immune cells (i.e., dendritic cells, natural killer cells, macrophages), induction of epigenetic modifications via histone acetylation/methylation in tissues [139] and through the production of signaling molecules including SCFA and bile acids. For example, the anti-inflammatory and immuno-modulatory effects of SCFAs are mediated through the differential activation of G protein coupled receptors (GPCRs) including GPR109a, GPR41 and GPR43 [140]. While bile acids differentially activate FXR and TGR5, which attenuate pro-inflammatory innate immune response in several autoimmune diseases including IBD [141].

Microbial sensing by the host immune system is achieved due to the presence of pattern recognition receptors (PRRs), which recognize conserved microbe-associated molecular patterns on bacteria. These PRRs include toll-like receptors (TLRs) and nucleotide oligomerization domain like receptors (NLRs), present on both immune and non-immune cells. These play a key role in the recognition of extracellular and intracellular bacteria and control the inflammatory response [142]. Ligand activation of NLRs and TLRs and their downstream signaling pathways ultimately lead to the expression of inflammatory cytokines and antimicrobial molecules [143]. Dysbiosis is frequently found in the GI systems of humans that exhibit chronic inflammation and this may play a critical role in shaping the composition of microbiota and the resulting dysbiosis [144].

A range of approaches are available to assess the host immune system including the measurement of cytokines and chemokines, gene expression and/or protein expression (reviewed [145]). Cytokines are one of the most common targets to be measured. Interleukin-1β (IL-1β) is a potent pro-inflammatory cytokine that exerts a range of systemic and local effects including promotion of immune cell recruitment to the site of inflammation; activation of dendritic cells, macrophages and neutrophils [146]. A variety of other inflammatory biomarkers can be measured. In a metanalysis on the effect of probiotic supplementation on inflammatory biomarkers in adults (42 randomized clinical trials, 2258 adults) a significant reduction was reported in serum C-reactive protein (CRP), tumor necrosis factor-α (TNF-α), IL-6, IL-12 and IL-4 concentrations [147]. However, a meta-analysis focusing on the elderly (10 randomized controlled studies, 689 elderly individuals) did not demonstrate any significant benefit of microbiota-driven therapy in decreasing the inflammatory responses of elderly individuals to a range of inflammatory markers including TNF-α, IL-6, IL-10, CRP, IL-1β, IL-l8 or MCp 1 [148].

Traditionally, the single-plex ELISA has been used in the assessment of cytokines and chemokines. However, despite the accuracy and value of this technology it precludes the capacity to investigate cytokine network interactions. In contrast, multiplex immunoassays simultaneously measure multiple biomarkers, including a large number of cytokines and chemokines, using minimal sample volumes allowing multifaceted immune responses to be studied [149, 150]. Another commonly used approach in human studies is qPCR [151]. This technique allows cytokine expression (mRNA) and pattern recognition receptors (TLRs, NLRs) to be analyzed across a wide range of tissues. Importantly, minimum information for publication of quantitative real-time PCR experiments guidelines should be followed when using such approaches [152].

Regardless of the approach taken to investigate microbiota–host immunological interactions, the type(s) of sample collected are crucial. Peripheral blood samples are reflective of systemic immune effects but less so the colon. In contrast, fecal samples are more representative of the colonic environment but do not fully reflect the events within the mucosal tissue. From a microbiota perspective, there are two distinct ecosystems in the colon both with different metabolic and immunological functions. This includes the luminal microbiota, accessible from fecal samples and representing the ‘critical mass’ for dietary and other metabolic conversions, and the mucosal-associated microbiota, directly interacting with the intestinal immune system and accessible from tissue biopsies [153]. To date, most gut microbiota studies have assessed microbial diversity through analysis of the luminal microbiota which poorly reflects microbial diversity at the mucosal surface [154, 155]. Mucosal biopsies exhibit more microbial diversity and pronounced differences in the dominant bacterial populations than fecal samples [154]. They are considered the “gold standard” for studying the crosstalk between mucosal associated microbes, the intestinal epithelium and tissue-specific immune responses. However, mucosal biopsies are surgically invasive, requiring specialist facilities for collection, and provide limited quantities of tissue restricting multi-omics based analysis of gut microbiota function [59]. Additionally, the bowel cleansing preparation used prior to colonoscopy (and biopsy collection) alters the composition of both the luminal and mucosal gut microbiota [156–158] and molecular targets in tissue [159, 160]. As such, biopsies are rarely collected in studies focused on dietary interventions in healthy humans. Although fecal samples appropriately collected [66, 161] are less reflective of the mucosal microbiota–host interaction, they have an advantage in terms of quantity of material available, convenience of sampling and arguably a wider applicability. Multiple sampling points are recommended in studies including baseline, early, middle and late time points, if possible [145]. Careful selection of sample collection (preservatives, additives used), preparation methods for processing tissue, sample storage and the number of freeze/thaw cycles (preferably a single cycle) is required as these can directly impact on the measured outcomes [162–165].

### Assessing host outcomes

The parameters measured are determined by the central research question and over-arching hypothesis. This can include measures such as body weight, BMI, fat mass and fat percentage or physiological measures, such as, blood pressure (BP), cognitive function and appetite.

There are numerous metrics proposed to reflect different aspects of gut health such as transit time, and stool volume and frequency. However, many measures are indicative of poor or impaired gut health rather than gut health per se such as bloating, abdominal cramps and diarrhea [166]. Measures of gut permeability are promoted as markers of gut health. Sugar tests (mannitol, lactulose, rhamnose) are commonly used but their accuracy is questionable and their potential to act as substrate for the gut microbiota raises questions over their validity. Other markers of barrier function include fecal A1 anti-trypsin and Reg-1 and plasma lipopolysaccharide. Plasma concentrations of intestinal-fatty acid binding protein provide information on gut damage while fecal myeloperoxidase and neopterin can be used as markers for intestinal inflammation.

### Statistical analysis plan

Except for exploratory trials, the statistical hypothesis and principal features of data analysis of the primary variable(s) should be specified at the stage of study preparation and before submission of the protocol to ethics and/or authorities. This should be in the form of a statistical section of the protocol. Also, a Statistical Analysis Plan (SAP) describing the detailed procedures for executing the statistical analysis of the primary and secondary variables may be written after finalizing the protocol. The SAP should be reviewed and possibly updated as a result of the blind review of the data and agreed between parties (Sponsor and Principal Investigator) before breaking the blind with formal records of SAP finalization date and unblinding date. If a microbial-related variable(s) has been selected as a primary or secondary endpoint(s) of the clinical study, the methodology for analysis cannot be changed after breaking the blind. Further analysis could be done as exploratory only. All these procedures ensure validity of the data. Failing to specify in the study protocol the information on who performs the analysis and where it is to be performed may greatly restrict options for ethical reasons. High-quality journals will also likely require proof that the SAP was conceived before study analysis in addition to the requirement of the study protocol deposition. Most high-quality journals require deposition of raw sequencing data and access to all relevant data. Code relating to the statistical tools used in the analysis should also be publicly available where possible. The above requirements may sometimes clash with the need to protect personal data. In this regard, it is important to note that all microbiota-related studies must be compliant with the new GDPR in Europe (see section on legislative aspects).

### Reporting of adverse events

In all clinical trials adverse effects are systematically monitored as part of good clinical practice (GCP). The safety profiles of nutritional interventions are generally considered very good when approved ingredients are used in the intervention.

## Legislative aspects in relation to foods and health claims in EU

One common objective of conducting human microbiota food interventions is in support of food related health claims. From a regulatory perspective in the European Union (EU), ‘Food’ is defined as any substance or product, whether processed, partially processed or unprocessed, intended to be, or reasonably expected to be ingested by humans. Thus ‘Food’ includes drink, chewing gum and any substance, including water, intentionally incorporated into food during its manufacture, preparation or treatment. There are many different classifications of foods/food ingredients in EU (for example, conventional food, food supplements, novel foods, foods for special medical purposes, food additives) and each of these serves a specific intended purpose and is regulated accordingly. The key general principles and requirements of food law and the general provisions relating to the labelling, presentation and advertising of foodstuffs are outlined in legislation [167, 168].

Food, when incorporated into the diet generally provides a source of energy and nutrition and may promote health, reduce the risk of developing a disease or manage a disease by dietary means. In contrast, a medicinal product is ‘any substance or combination of substances presented as having properties for treating or preventing disease in human beings; or any substance or combination of substances which may be used in or administered to human beings either with a view to restoring, correcting or modifying physiological functions by exerting a pharmacological, immunological or metabolic action, or to making a medical diagnosis’ [169]. Thus, even a ‘conventional food’ presenting with associated claims relating to preventing or treating a disease would be classified as a medicinal product even if the efficacy data supported the claims.

Live microorganisms, such as, probiotic bacteria are considered food ingredients and depending on their history of use may be considered novel ingredients in EU. For example, any food consisting of, isolated from or produced from microorganisms or fungi and that has not been consumed to a significant degree within the EU before 15 May 1997 would be considered novel. In all there are ten novel food categories to consider when determining if a food/food ingredient is novel or not. For microorganisms that have a history of use, a harmonised pre-market risk assessment approach exists—“Qualified Presumption of Safety” (QPS). This approach considers if the safety assessment of a defined taxonomic group (e.g., genus or group of related species) can be made based on their identity, body of knowledge, possible pathogenicity and end use. If the taxonomic group does not raise safety concerns or, if safety concerns that exist can be defined and excluded, the grouping can be granted QPS status. Any strain of microorganism the identity of which is unambiguously established and assigned to a QPS group is free from the need for further safety assessment other than satisfying any qualifications specified. While many microorganisms are considered QPS, novel microorganisms must undergo evaluation by the European Food Safety Authority (EFSA) before being placed on the market to ensure safety [170]. An example of a probiotic bacteria authorized as a novel food ingredient in the EU is *Clostridium butyricum MIYAIRI 588*.

When planning food studies in subjects, in addition to food safety requirements, The Helsinki Declaration, which sets out guiding principles for the ethical conduct of human studies, GCP Guidelines and the requirements of local ethical review boards should be taken into consideration [171, 172]. In addition, registration of trial details in a publicly accessible registry is considered best practice and is a requirement of many Journals for publication. With the introduction of the Clinical Trial Regulation in 2019, the conduct of clinical trials on medicinal products in the EU will undergo a major change [173]. The assessment and supervision processes will be harmonized via an EU portal and a database managed by the European Medicine Agency, while the authorization and oversight of clinical trials will remain the responsibility of Member States. Ensuring correct classification of any new product in the development phase is important before any human trials are planned to ensure regulatory compliance. Food business operators (FBO) involved in conducting nutrition/food trials involving the microbiota also need to be aware of the compliance requirements outlined in the new General Data Protection Regulation (GDPR) which is designed to enable individuals to better control their personal data—‘any information relating to a person who can be identified, directly or indirectly, in particular, by reference to an identifier such as a name, an identification number, location data, online identifier or to one or more factors specific to the physical, physiological, genetic, mental, economic, cultural or social identity of that person’ [174]. Furthermore, under GDPR even organizations outside the EU that collect data concerning an EU resident are subject to the jurisdiction of the EU regulators.

The Nutrition and Health claims Regulation was the first piece of harmonized EU legislation to lay down specific rules governing the use of nutrition and health claims made on food. It lays down the types of claims, the rules on scientific substantiation and the routes for authorization of new health claims for foods [175]. It should be noted that specific rules apply to certain food classifications, for example, infant formula which is considered the only processed foodstuff fully satisfying the nutritional requirements of infants during the first month of life. Given this special status of infant formula, the use of health claims is prohibited ([176] applies Feb 2020). For follow-on formula (food for infants when appropriate complementary feeding is introduced), nutrition and health claims are allowed [176]. Also, in future there may be a possibility, following an EFSA assessment, where it is demonstrated that a specific formula manufactured from protein hydrolysates reduces the risk of developing allergy to milk proteins, that consideration will be given to how to adequately inform consumers about that property of the product. Evaluation of health claims is carried-out by the EFSA and to date more than 3000 health claims have been evaluated, the majority of which were not considered to be scientifically substantiated [177]. A large number of probiotic-related health claims were among these rejections reflecting short-comings in the quality of submissions in the main. Key areas where issues were raised in relation to probiotic-related health claims were insufficient characterization of the ingredients, lack of demonstration that the claimed effect was beneficial to health and demonstration that a cause and effect relationship existed and was supported by high quality pertinent studies. For the substantiation of a health claim, the choice of an appropriate outcome variable(s) and method of measurement(s) are critical. EFSA scientific guides and published opinions are a valuable source of such information, highlighting beneficial physiological effects and outcome variables which could be acceptable for claim substantiation, or to address potential health relationships. For example, EFSA guidance indicates that GI discomfort may be measured using validated subjective global symptom questionnaires and changes in one or more of the individual symptoms as well as changes in defecation habits may be used as supportive evidence for the mechanisms by which the food could exert the claimed effect. However, these cannot be used alone for the substantiation of a claim on the reduction of GI discomfort. Some of the effects not considered beneficial per se by EFSA were, increasing the number of any group of microorganisms, changes in short-chain fatty acid production or pH and stimulation of various immunological responses [178]. Thus, particular care should be taken at the study design phase to ensure that the outcome variables chosen to substantiate an effect is indeed considered valid. In EU, the statement “contains probiotics/prebiotics” is of itself considered as a health claim [179] though noteworthy, Italy allows, ‘able to support the balance of intestinal flora’, ‘probiotics’ and ‘prebiotics’, to be used on labels as in their assessment these are not health claims [180]. Claims in advertising regarding bacteria which are not nutrition or health claims may be dealt with under the general rules against misleading advertising. Thus, if a statement regarding bacteria does not refer to a nutritional benefit or a relationship between a food or ingredient and health, the FBO should still hold evidence to substantiate the claim. While authorization of health claims related to the benefits of the intake of specific microorganisms remains challenging, updated EFSA guidance and opinions provide valuable guide rails in relation to study design and required documentation. Arguably, pre-submission dialogue with EFSA to address the design of trials and submission requirements would be invaluable to alter the *status quo* on probiotic health claim authorization in EU. A key recommendation is to consider/determine the regulatory classification of the proposed intervention in the planning phase, in advance of human trials to ensure regulatory compliance.

## Conclusion

As the influence of the intestinal microbiota on human health becomes more apparent, there is an increasing motivation to develop and evaluate nutritional interventions to manipulate this microbial-human relationship to improve health. In this review we outline important considerations when designing and interpreting human studies to reliably establish such links. This includes appropriate study design, intervention and control selection, participant criteria and the appropriate outcome measures to demonstrate tangible effects of the intervention of host health. With the rapid and continuing evolution of the field, both in terms of understanding and the tools available to measure microbial–host interactions, the list of outcome measures presented is not exhaustive nor static. Care must be taken to avoid bias at all levels of the study and negative as well as positive outcomes should be reported for transparency and confidence.

## Data Availability

Data sharing not applicable to this article as no datasets were generated or analysed during the current study.

## Abbreviations

AHR: Aryl hydrocarbon receptor
ANOVA: Analysis of variance
BMI: Body mass index
BP: Blood pressure
CRP: C-reactive protein
EFSA: European Food Standards Agency
FBO: Food business operators
FISH: Fluorescence in-situ hybridization
FXR: Farnesoid-X-receptor
GC: Gas chromatography
GDPR: General data protection regulation
GPCRs: G-protein coupled receptors
IBD: Inflammatory bowel disease
IBS: Irritable bowel syndrome
IL: Interleukin
LC–MS: Liquid chromatography-mass spectrometry
LPS: Lipopolysaccharide
MA: Methylamine
mRNA: Messenger RNA
mTORC1: Mechanistic target of rapamycin complex 1
NEPP: Non-extractable polyphenols
NF-κB: Nuclear factor-κB
NGS: Next generation sequencing
NMR: Nuclear magnetic resonance
NOD: Nucleotide oligomerization domain
NLR: NOD-like receptors
OTU: Operational taxonomic units
permANOVA: Permutational ANOVA
PRRs: Pattern recognition receptors
qPCR: Quantitative polymerase chain reaction
QPS: Qualified presumption of safety
RCT: Randomized controlled trial
SAP: Statistical analysis plan
SCFA: Short-chain fatty acids
SSU rRNA: Small subunit ribosomal RNA
TLR: Toll-like receptors
TMA: Trimethylamine
TMAO: Trimethylamine*-N*-oxide
TNF-α: Tumor necrosis factor-α
